# The effectiveness and user preferences of two tactile breathing devices in reducing stress in stressed individuals: A mixed methods study

**DOI:** 10.1016/j.ijchp.2025.100603

**Published:** 2025-07-09

**Authors:** E. Honinx, M. Meys, S. Broes, L. Van Langenhoven, R. Janssens, I. Huys, V. Oswald, J. Annen, S. Laureys, C. Martial, O. Gosseries

**Affiliations:** a Moonbird, Antwerp, Belgium; b Coma Science Group, GIGA-Consciousness, University of Liège, Belgium; c Centre du Cerveau, University Hospital of Liège, Belgium; d KU Leuven - University of Leuven, I-BioStat, Leuven B-3000, Belgium; e KU Leuven - University of Leuven, Clinical Pharmacology and Pharmacotherapy, Leuven B-3000, Belgium; f Cognitive and Computational Neuroscience Lab, Psychology Département, University of Montréal, Montreal, Canada; g CERVO Research Institute, Laval University, Quebec, Canada

**Keywords:** Stress management, Breathing devices, User preferences, Physiological measures, EEG, ECG, Respiration

## Abstract

**Background:**

Rising stress levels have led to increased interest in stress management tools, particularly tactile breathing devices. Despite their popularity, there is limited evidence on their physiological and psychological effectiveness and user perceptions. This study evaluates the effectiveness of and preferences toward two tactile breathing devices among highly stressed individuals.

**Methods:**

The study involved 36 participants using two breathing devices, moonbird and Core. Physiological data were collected using EEG, ECG, and a breathing belt. User preferences and self-reported experiences were assessed via questionnaires.

**Results:**

Moonbird usage was associated with increased delta power and decreased alpha power, while Core did not significantly modify EEG power. ECG analysis indicated no significant differences in mean heart rate between devices. Both devices reduced heart rate variability during use, but no lasting effects were observed post-intervention. Respiratory rates decreased during both devices’ use, with moonbird showing more sustained effects post-intervention. There were no significant differences in self-reported relaxation and energy levels between the devices, though moonbird was preferred overall for its handling and breathing guidance.

**Conclusion:**

Both devices demonstrated the ability to lower physiological stress, as indicated by improvements in certain neurophysiological measures during use, with moonbird preferred for its ergonomic design and tactile feedback. These findings underscore the importance of user experience in device effectiveness, highlighting the need for a user-centric approach in device design. Future research should explore long-term effectiveness, real-world user feedback, and the physiological and psychological mechanisms associated with these devices.

## Introduction

Stress affects about 40 % of the global population, significantly impacting mental and physical health (Gallup, 2023; Nochaiwong et al., 2021; Mahmud et al., 2021; WHO, 2023). It carries societal and financial consequences, increasing the burden on healthcare systems (Cigna, 2019). This underscores the urgent need for effective management strategies. Traditional approaches often rely on pharmacological treatments, for which improvements are limited and can come with side effects and tolerance (Leichsenring et al., 2022; Noel et al., 2002). Recently, non-pharmacological methods like meditation, physical exercise, and mindfulness have gained interest (Anthierens et al., 2010). Among these, breathing exercises stand out for their potential in stress alleviation by altering respiration patterns to enhance well-being (Fincham et al., 2023).

Slow breathing techniques, characterized by a rate of 5–8 breaths per minute, have been shown to positively influence the autonomic nervous system (ANS), particularly the balance between its sympathetic and parasympathetic branches (Lehrer, 2018; Zaccaro et al., 2018). The parasympathetic nervous system, acting as the body's brake pedal, promotes physiological and psychological relaxation when breathing slows to around 6 breaths per minute.

The activation of the parasympathetic nervous system induces physiological reactions such as decreased heart rate and breathing rate. Another crucial measure of parasympathetic activity is heart rate variability (HRV), which reflects the variability between heartbeats (Rajendra Acharya et al., 2006). Higher HRV indicates better stress adaptability, while low HRV is linked to stress-related disorders (Gerbarg et al., 2019). Slow breathing has been shown to increase HRV, promoting relaxation and reducing stress responses (Taylor et al., 2001; Pramanik et al., 2009; Soer et al., 2021).

EEG measurements reveal that slow breathing can shift brainwave patterns from high-frequency beta waves, associated with stress, to more relaxed alpha and theta waves (Jerath et al., 2006). This change suggests a state of mental relaxation and improved emotional regulation. EEG data also indicate increased coherence in brain activity, linked to cognitive function enhancements (Fincham et al., 2023; Heck et al., 2022).

Breathing exercises can also promote psychological relaxation, improving emotional well-being and resilience. Studies indicate that guided breathing techniques can reduce perceived stress and anxiety, enhancing mood and overall mental clarity (Balban et al., 2023). The combination of physiological and psychological benefits makes breathing exercises a powerful tool for holistic stress management.

However, the effectiveness of breathing exercises can vary based on technique, motivation, and consistency (Tavoian et al., 2023). Research suggests that guided exercises, provided by a coach or an app, are more effective in increasing relaxation than self-guided techniques, which require individuals to self-regulate (Sharpe et al., 2021). In response to this, there has been a rise in the development of breathing guidance devices, including apps, smartwatches, breath pacers, and audio guides (Honinx et al., 2023). These devices often employ tactile, visual, or auditory stimuli to guide users into a slower breathing rhythm (Honinx et al., 2023). Despite the growing availability of such tools, their effectiveness may vary depending on the type of stimuli and user preferences (Honinx et al., 2023; Yu et al., 2015; Busch et al., 2012).

A recent review on stress-reducing devices highlights a mismatch between current solutions and user needs, as device development often lacks input from end-users, affecting uptake and effectiveness (Honinx et al., 2023). Previous unpublished user studies have shown preference for tactile stimulation, such as vibration or expansion/contraction, over other stimuli. In addition, research has shown that users experience over 40 % improvement in performance during breathing therapy when haptic feedback is utilized (Janidarmian et al., 2016). Participants have also emphasized the importance of devices being handheld and portable. These insights have informed the design of this study, which examines the effectiveness and user preferences for two tactile, handheld breathing devices—the Core meditation trainer (Hyperice©) and the moonbird breath pacer (Moonbird© BV)—in combination with calming music.

This study specifically assesses: (i) the effectiveness of these devices, as indicated by changes in heart rate, breathing rhythm, and EEG; (ii) user preferences regarding the devices and their features; and (iii) user-reported impacts on stress.

We hypothesize that (1) device-guided slow breathing will lead to physiological stress reduction, assessed through heart rate, breathing rhythm, and EEG; (2) there will be a distinct user preference for one of the devices; (3) device-guided breathing will also result in psychological stress reduction, assessed through questionnaires. Additionally, we anticipate (4) stress reduction effects will vary between devices, and (5) these variations will correlate with user preference.

This study aims to contribute to user-centered, evidence-based approaches in stress management by highlighting the importance of aligning device design with user experiences and preferences, ultimately promoting their effectiveness and uptake in reducing stress.

## Methods

### Design and recruitment

This prospective cross-over study used a mixed method design including (1) a quantitative assessment of the effectiveness of two tactile breathing devices for stress reduction in stressed individuals using an electrocardiogram (ECG), a breathing belt, and a high-density EEG; and (2) a qualitative assessment of participant preferences and effects using standardized questionnaires and a visual analog scale (VAS).

We recruited participants between 18 and 65 years through email, social media, and posters. Only individuals who scored moderate to high (i.e., 27–56) on the Perceived Stress Scale (PSS-14) were included (Cohen et al., 1983). Exclusion criteria encompassed a history of physical disability or mental disorders (aside from stress) and the use of prescription medication.

Those who expressed interest were able to register using an online screening form (Qualtrics, 2023). This form included comprehensive information about the study, data processing, informed consent, as well as socio-demographic questions on age, gender, educational background, language proficiency, medical history, and current medication usage. Participants also completed the PSS-14.

### Materials

**Fig. 1:**
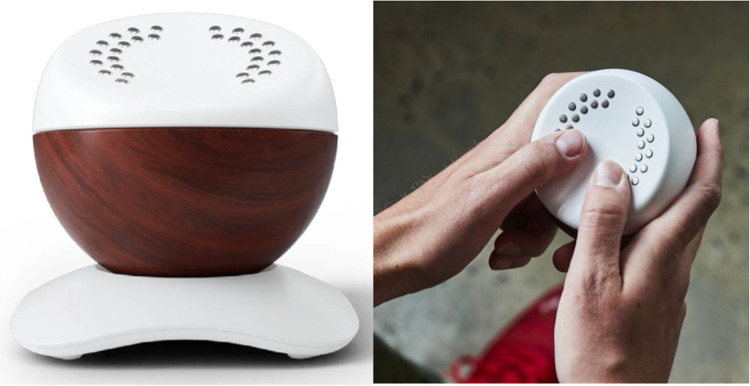
The Core breathing device.

**Fig. 2:**
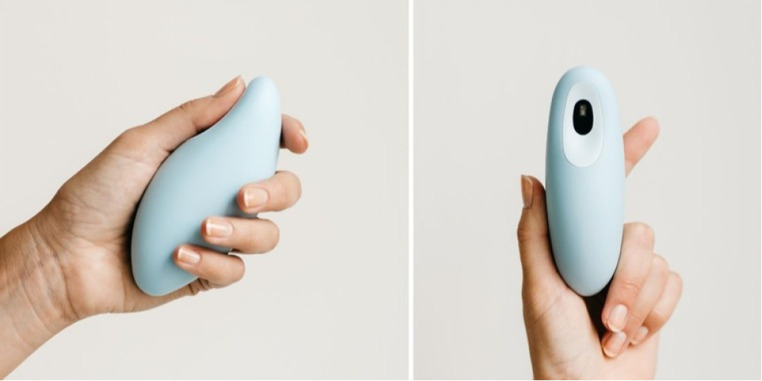
The moonbird breathing device.

Based on our previous unpublished work (i.e., an online survey with 500 respondents and focus groups with 30 participants), two breathing devices were chosen for this study. The first device, the Core meditation trainer (Hyperice©), is a spherical object measuring 89 × 89 × 89 mm (Fig. 1). It employs dynamic pulses and lights to guide the user's breathing rhythm. Users hold the device with both hands, touching the 2-electrode electrocardiogram, meanwhile inhaling as the vibrations and lights intensify and exhaling as they fade. The moonbird device (Moonbird© BV), reviewed in prior studies, was selected as the second device (Vermeylen et al., 2022). Moonbird’s dimensions are 122 × 54 × 44 mm, and the device is encased in biocompatible, medical-grade silicone. Designed for tactile breathing guidance, it physically expands and contracts to a slow rhythm, encouraging users to synchronize their breath accordingly (Fig. 2). Users hold the device, touching the photoplethysmogram sensor, activated when the thumb contacts the sensor. They inhale as the device expands and exhale as it contracts.

ECG, respiration, and EEG were simultaneously acquired throughout the experiment using an actiCHamp Plus system at a sampling rate of 1000 Hz (Brain Products GmbH, 2019). The ECG setup approximated a type II lead with electrodes at the sternum and ribs. Respiratory data were acquired with two respiratory inductance plethysmography sensor belts placed around the thorax and abdomen (Philips Respironics, 2023). The EEG montage consisted of a 128-channel R-Net layout, applied with central electrode Cz as online reference and sponges soaked in a potassium chloride solution.

### Procedure

Upon arrival at the GIGA Institute, participants were asked to submit their completed informed consent form or read and complete them on-site. Subsequently, the researcher provided a concise overview of the study procedure, allowing time for questions. No manufacturers or device brands were disclosed. Head measurements were taken to determine the appropriate size of the EEG cap (S, M, L). The participant was then connected to the ECG, respiratory belt, and EEG apparatus to accurately measure heart rate, breathing rate (via chest movements), and brain activity, respectively.

**Fig. 3:**
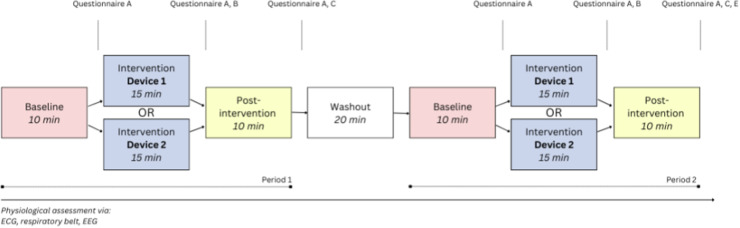
Protocol of the study. Questionnaire A: general experience, Questionnaire B: device use and experience, Questionnaire C: after-effects device use, Questionnaire E: device preferences.

The experiment was divided into three phases (Fig. 3): a baseline (10 min), an intervention phase during which the participants used the device (15 min), and a post-intervention resting phase (10 min). Both during the baseline phase and the post-intervention resting phase, participants sat in silence and were instructed to breathe at a normal rate (resting state recording). During the intervention phase, participants were instructed to breathe along with one of the two breathing devices, following a guided 4–8 exercise (inhaling for 4 s, exhaling for 8 s). During all three phases, relaxing music (by *Tranquil Relax*) was played in the background, and participants were asked to close their eyes.

Participants had no prior experience with either device. During the intervention, they were instructed by an experienced researcher on how to properly hold each device (Core with both hands, moonbird with one hand) and how to place their fingers on the device sensors to activate the devices. Before beginning the exercise, they had a chance to familiarize themselves with the device, during which the researcher ensured they held it correctly, understood the guidance, and could follow the indicated breathing rhythm. Additional guidance was provided if needed.

The participants first underwent the three phases with one device (Period 1), and then, after a washout period of 20 min, again with the second device (Period 2). This resulted in seven phases in total (Fig. 3). The duration of the experiment was 90 min. The order in which devices were presented to participants was randomized, ensuring half began with moonbird and the other half with Core. During the study, participants completed several questionnaires to assess their experiences and the effects of the breathing devices. After each of the phases, except the washout phase, participants filled out Questionnaire A, which focused on their general experience. Following both intervention phases, participants completed Questionnaire B to evaluate device use and experience. After each post-intervention resting phase, they filled out Questionnaire C, which assessed the after-effects of using the device. At the end of the experiment, participants completed Questionnaire E to express their preferences towards the devices.

A debriefing session concluded the study, addressing any questions. Participants received monetary compensation of 45 euros for their participation.

### Questionnaires

#### Questionnaire A (General experience)

*Experience of the intervention:* Participants were asked to answer 5 questions with a VAS: “Could you estimate on a 0 (completely untrue) to 10 (completely true) scale if, during the phase you have just experienced, you experienced the following: the ability to clear your head; a well-rested feeling; more energy; a direct relaxing effect; and a negative effect.”

*Level of sleepiness*: Participants were presented with the validated Karolinska sleepiness scale (Kaida et al., 2006). This 9-point scale (ranging from “Extremely alert” to “Very sleepy, I had to make a lot of effort to stay alert; I was fighting sleep”) measures the subjective level of situational sleepiness. On this scale, participants indicated which level best reflects their psycho-physical state experienced in the last 10–15 min (during the preceding phase).

*Level of absorption*: Participants were required to fill in a VAS regarding the level of absorption (Vanhaudenhuyse et al., 2019). They were asked the following question “Could you estimate on a 0 to 10 scale how deeply you felt absorbed and felt your attention focused on the phase you have just experienced? 0 means you were not absorbed at all in the experience; 10 means you were fully absorbed in the experience.”

*Level of dissociation:* Participants were required to fill in a VAS regarding the level of dissociation (Vanhaudenhuyse et al., 2019). They were asked the following question: “Could you estimate on a 0 to 10 scale if you felt a dissociation between your bodily sensations and the actual environment? 0 means you were in this ‘reality’, in this room; 10 means that you completely escaped in your subjective experience, totally disconnected from the here‐and‐now reality.”

*Time perception:* Participants were asked to estimate the duration (in minutes) of each phase.

#### Questionnaire B (Device use and experience)

Participants were asked the following 10 questions: “Could you estimate on a 0 (completely untrue) to 10 (completely true) scale if, with regard to the device: the device was easy to use; the device was easy to hold in your hand; the device gave a pleasant stimulation; the device was easy to follow; the device had a relaxing effect; the combined music was pleasant; the combined music was relaxing; you would use it again; you would buy it; you would recommend it to others.”

#### Questionnaire C (After-effects)

This questionnaire included the validated Relaxation State Questionnaire (RSQ) for evaluating momentary relaxation (Steghaus et al., 2022). Participants were asked the following 10 questions: “Could you estimate on a 1 (completely untrue) to 5 (completely true) scale if, after using the device compared to before, you experienced the following: rapid breathing; increased heart rate; tense muscles; relaxed muscles; loose muscles; relaxed feeling; calm feeling; feeling sleepy and tired; falling asleep; feeling refreshed and awake.”

#### Questionnaire E (Preference)

Participants were asked to express their preferences (Yes/No/No answer) and reasoning regarding the two breathing devices. Specifically, they were questioned about their *general preference* for one of the devices. Additionally, participants were asked about their *preference* in terms of *how the device should be held* and in terms of *how it guides their breathing* (through vibration or expansion/contraction).

All questionnaires were presented via Qualtrics.

### Ethics

The study received approval from the Ethics Committee of the Faculty of Medicine at the University of Liège (2022–66), complying with GDPR and the 1964 Helsinki Declaration. Participants signed informed consent forms detailing the study's objectives and methods. They were informed orally and in writing about their right to withdraw at any time. The study had insurance coverage for accidental damages. Data confidentiality was ensured with identification numbers known only to investigators. Physiological data were securely stored at the Coma Science Group, protected by a firewall, while questionnaires were managed using the secure Qualtrics platform. Only responsible researchers maintained personal data.

### Data processing and analysis

ECG and respiratory signals were processed and analyzed in Python using the Neurokit2 toolbox for biophysiological data analysis (Makowski et al., 2021). ECG signals were cleaned by applying a fifth order 0.5 Hz high-pass Butterworth filter and removal of the 50 Hz power line interference. Individual heartbeats were identified using the default Neurokit2 peak detection algorithm as R peaks from corresponding QRS complexes (as verified in Brammer, 2020). The resulting peaks were carefully visually inspected, after which missing and superfluous instances were manually added or removed. The mean heart rate was subsequently derived and heart rate variability (HRV) measures were extracted from RR intervals. Measures characterizing the variation between successive heart beats in the time domain include the standard deviation of RR intervals (SD NN), the root mean square of successive differences between adjacent RR intervals (RMSSD NN), and the proportion of successive RR intervals that differ >50 ms (pNN50). Frequency domain measures consist of normalized low frequency spectral power (LFn, 0.04 to 0.15 Hz), high frequency spectral power (HFn, 0.15 to 0.4 Hz), and the ratio of low to high frequency spectral power (LF/HF). Approximate entropy (ApEn), a method for measuring the regularity and unpredictability of heart rate in a time series, was considered as a nonlinear measure of HRV complexity. HRV measures that were significantly correlated with the heart rate were corrected before applying statistical analyses through multiplication with or division by the mean RR interval duration up to an appropriate power, depending on the direction and strength of this dependency (Sacha, 2013, 2014; Sacha et al., 2013).

Respiratory signals were cleaned by applying a second order 0.05–3 Hz band-pass Butterworth filter. Extrema of respiration were determined based on the maxima and minima between zero-crossings, resulting in pairs of peaks (expiration onsets) and troughs (inspiration onsets) (Khodadad et al., 2018). The resulting values were carefully visually inspected and manually corrected where necessary. Measures derived directly from the breathing signals include the mean respiratory rate and the phase duration ratio (PDR), the latter being the ratio of inspiratory to expiratory time. F or respiratory rate variability (RRV) measures, similarly to their HRV counterparts but with breath-to-breath (BB) cycles rather than RR, we considered the standard deviation of the BB intervals (SD BB) and the root mean square of successive differences between adjacent BB intervals (RMSSD BB).

EEG signals were processed and analyzed in MATLAB using a semiautomated pipeline (https://github.com/srivaschennu/MOHAWK), as introduced in Chennu et al. (2014), based on functionalities from the EEGLAB and FieldTrip toolboxes (Delorme et al., 2004; Andersen, 2018). The raw EEG was down-sampled to 250 Hz, bandpass filtered between 0.5 and 45 Hz (Hamming windowed sinc finite impulse response filter), and segmented into 10-second epochs, which were subsequently baseline corrected. Noisy channels and epochs were manually marked and rejected from further processing. The resulting data was cleaned using independent component analysis to remove signal components corresponding to non-neural sources (i.e., eye movements, muscular activity). Rejected channels were finally reconstructed through spherical spline interpolation and the data was re-referenced to the average over all channels.

Power spectral and connectivity measures were obtained as the output of the same pipeline used for processing (https://github.com/srivaschennu/MOHAWK), with default parameters and no modifications. Following this approach, channel-wise power spectra were estimated by applying a multiple-taper fast Fourier transform. Relative power values were then obtained as the contributions of the five canonical frequency bands (delta: 0.5–4 Hz; theta: 4–8 Hz; alpha: 8–13 Hz; beta: 13–30 Hz; gamma: 30–45 Hz) to the total power. Brain connectivity was computed as the debiased weighted phase lag index (dwPLI), a measure that reflects phase synchronization between signals, these being EEG traces measured at the different electrodes (Vinck et al., 2011). Resulting relative power values and symmetric 127 × 127 connectivity matrices obtained for each frequency band were subsequently averaged over all channels to obtain a global value for EEG power and connectivity, respectively. Lastly, the Lempel-Ziv complexity (LZC) was considered an index of regularity of the brain’s dynamics (Lempel et al., 1976). This measure was calculated using an implementation in MATLAB (Thai, 2024) with default parameters and for each epoch and channel individually, following binarization of the EEG signals’ magnitudes (absolute value of Hilbert transform) with the mean value over time as threshold. Resulting values were averaged over epochs and channels to obtain a global value.

### Statistical analysis

The primary focus of the analysis is on heart rate, HRV, breathing rate, EEG power, EEG connectivity, and EEG complexity during the intervention compared to baseline, and user preferences (general preference, device handling, and breathing guidance from Questionnaire E). Additionally, the analysis investigated (1) physiological measures: heart rate, breathing rate, EEG power, EEG connectivity, and EEG complexity during the post-intervention resting phase and differences between devices; and (2) the effect on and differences between devices for self-reported parameters: ability to clear head, well-rested feeling, energy level, direct relaxing effect, negative effect, and sleepiness (Questionnaire A); ease of use, ease to hold, guidance pleasantness, ease of following guidance, and relaxing effect (Questionnaire B); rapid breathing, increased heart rate, relaxed feeling, calm feeling, sleepiness or tiredness, and falling asleep (Questionnaire C). This subset of self-reported measures was selected for further analyses because they were the most relevant.

For physiological data, non-parametric tests were applied due to non-normality (Shapiro-Wilk tests). Measures during intervention and post-intervention phases were compared to baseline using Wilcoxon signed-rank tests with exact p-value calculations. Differences between device types were analyzed similarly, with Bonferroni corrections for multiple testing. The corrected significance thresholds were determined based on the number of statistical comparisons per modality, accounting for the repeated use of the baseline condition in both the intervention and post-intervention comparisons: *p* < 0.003125 for ECG (8 comparisons × 2 phases), *p* < 0.00625 for respiration (4 comparisons × 2 phases), and *p* < 0.005 for EEG (5 comparisons × 2 phases).

User preferences (Questionnaire E) were reported in frequencies and percentages. An exact binomial test assessed if one device was preferred over the other. For psychological stress reduction, within-period changes from baseline values (Questionnaire A) were analyzed using a mixed model with device and period as fixed effects and a random intercept for the subject. We present estimated effect sizes that are corrected for possible period effects with 95 % confidence intervals. Additionally, Bonferroni-corrections for multiple testing were also performed: *p* < 0.004166 (6 questions × 2 phases).

Differences between devices on self-reported data (Questionnaires A, B, and C) were calculated using the within-subject score difference (moonbird minus Core) and the Hill’s-Armitage approach for cross-over designs (Senn et al., 1993), correcting for period effects. Sensitivity analyses focused on first-period results to mitigate possible carry-over effects, using Mann-Whitney U tests. Additionally, Bonferroni-corrections for multiple testing were also performed: *p* < 0.004167 (6 questions × 2 phases) for Questionnaire A, *p* < 0.01 (5 questions × 1 phase) for Questionnaire B, and *p* < 0.00833 (6 questions × 1 phase) for Questionnaire C.

To assess differences in physiological stress reduction by preferred device (Questionnaire E), within-subject differences (preferred device minus the other) were calculated for intervention and rest phases, original values and adjusted for baseline. Summary statistics and appropriate paired samples *t*-tests, Wilcoxon signed rank tests, or Sign tests were used, categorized by preference type (General, Use, Guidance). Additionally, Bonferroni-corrections for multiple testing were also performed: *p* < 0.000298 (14 physiological stress parameters × 3 preference types × 2 phases × 2 adjusted for baseline or not). Analyses were performed using SAS 9.4 (© SAS Institute Inc.) and the SciPy Python library.

## Results

Our sample size was guided by similar prior research, which suggested a target of 20–25 participants (Rousseaux et al., 2023; Vondrasek et al., 2023). To account for technical issues (problems with measuring equipment) and participant cancellations, additional participants were recruited to ensure a minimum of 25 participants eligible for inclusion in the quantitative analyses. This resulted in 26 participants for heart rate, 25 for breathing rhythm, and 25 for EEG. Following completion of questionnaires, the final sample for the qualitative analyses consisted of 36 participants.

Of the 36 included participants, 69 % were female (Table 1). Most participants (94 %) were between 18 and 35 years old, two were older than 45 (6 %). The mean stress score as measured with the PSS-14 was 35, indicating a moderate to high level of stress (>37 is high). For half of the participants, moonbird was the first device used, the other half used the Core device first.

**Table 1:** Demographics of the participants per device order (Core - moonbird; moonbird - Core). Range of PSS-14 is 0–56.

**Demographics**	**Core - moonbird** ***N*=18**	**moonbird - Core** ***N*=18**	**Total** ***N*=36**
Gender F M	13/18 (72.22 %) 5/18 (27.78 %)	12/18 (66.67 %) 6/18 (33.33 %)	25/36 (69.44 %) 11/36 (30.56 %)
Age 18 - 35 years 45 - 65 years	18/18 (100 %) 0/18 (0 %)	8 16/18 (88.89 %) 2/18 (11.11 %)	34/36 (94.44 %) 2/36 (5.56 %)
Stress score mean (sd) min, max	35.4 (4.4) 27, 42	35.6 (5.3) 27, 44	35.4 (4.8) 27, 44

### Physiological stress reduction

Physiological data was included when all six recording phases were suitable, for the three signal types—ECG, respiration, EEG—independently (15 participants with all three, 12 with only two, 7 with only one, 2 with none). The intersection of participants included for ECG and respiration was 19, 21 for ECG and EEG, and 17 for respiration and EEG.

**Fig. 4:**
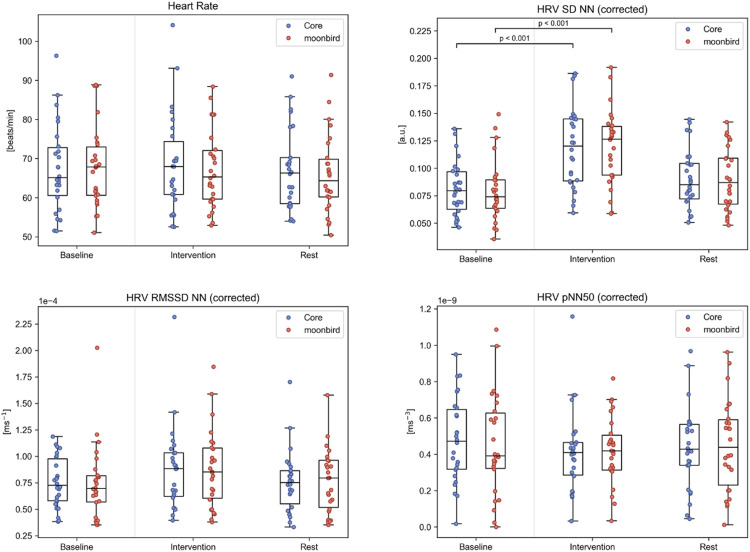
ECG and time domain HRV measures for the different phases and devices. HRV measures that were significantly correlated to the heart rate are depicted after having been corrected using the mean RR intervals.

**Fig. 5:**
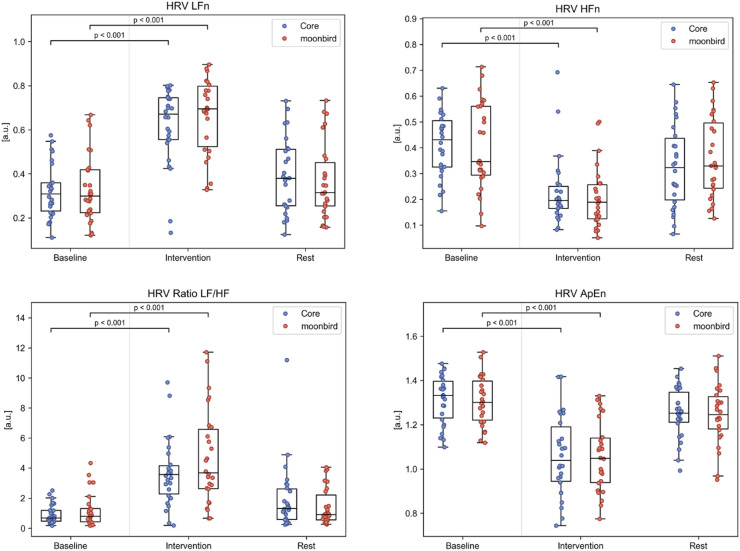
Frequency domain and complexity HRV measures for the different phases and devices. HRV measures that were significantly correlated to the heart rate are depicted after having been corrected using the mean RR intervals.

ECG recordings from 26 participants (13 with moonbird first) were used for heart rate and HRV analysis (Fig. 4, Table A.1). No significant differences in mean heart rate were found between any phases or devices. HRV SD NN was significantly higher during the intervention phase compared to baseline for both devices (median difference (Q1, Q3) Core: 0.034 (0.024, 0.052); moonbird: 0.036 (0.021, 0.049); *p* < 0.001). The other time domain HRV measures, RMSSD NN and pNN50, did not significantly differ for any phase or device. As for frequency domain HRV measures, LFn was significantly higher during the intervention phase compared to the baseline for both devices (median difference (Q1, Q3) Core: 0.34 (0.14, 0.45); moonbird: 0.36 (0.16, 0.48); *p* < 0.001), while HFn was significantly lower (median difference (Q1, Q3) Core: −0.20 (−0.33, −0.13); moonbird: −0.16 (−0.32, −0.06); *p* < 0.001) (Fig. 5). The consequent increase in LF/HF ratio reached significance as well for this same comparison and for both devices (median difference (Q1, Q3) Core: 2.51 (1.42, 3.76); moonbird: 3.32 (0.79, 5.32); *p* < 0.001). HRV ApEn was significantly lower during the intervention phase compared to baseline, again for both devices (median difference (Q1, Q3) Core: −0.26 (−0.42, −0.06); moonbird: −0.25 (−0.37, −0.16); *p* < 0.001). None of the ECG-derived measures showed any significant differences when comparing the post-intervention resting phase to the baseline following correction for multiple testing. No significant differences between devices were apparent.

**Fig. 6:**
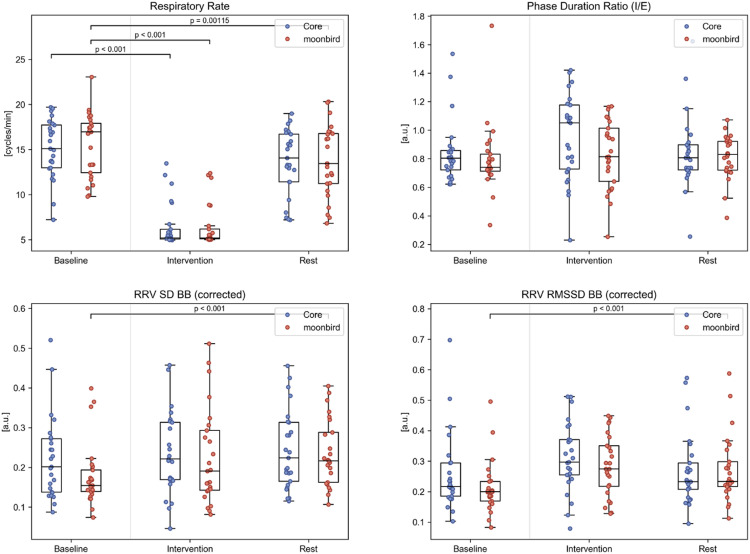
Respiration and RVV measures for the different phases and devices. RVV measures that were significantly correlated to the respiratory rate are depicted after having been corrected using the mean breath-to-breath (BB) intervals.

Thoracic respiratory signals from 25 participants (13 with moonbird first) were further analyzed (Fig. 6, Table A.2). The mean respiratory rate was significantly lower during the intervention phase compared to baseline for both devices (median difference (Q1, Q3) Core: −8.82 (−11.93, −6.10); moonbird: −8.30 (−12.38, −5.54); *p* < 0.001). However, in one out of five participants, the target of five breaths per minute as indicated by the devices could not be consistently reached, despite a relative slowing of respiration still manifesting. Four participants failed to achieve this exact desired effect with both devices, while the two remaining instances occurred during either the Core or the moonbird device use only. Mean respiratory rates in the resting phase also decreased compared to baseline for both devices, albeit only significantly for moonbird following the correction for multiple testing (median difference (Q1, Q3): −1.59 (−2.75, −0.07); *p* < 0.01). No significant differences in PDR between phases or devices were found. Both RRV SD BB and RMSSD BB tended to behave in an inversely proportional manner to the mean respiratory rate, to which they were significantly correlated. This dependency was removed by dividing both measures by their corresponding mean breath-to-breath intervals and, following this correction, the increases in SD BB and RMSSD BB in the resting phase compared to baseline remained significant for moonbird only (median difference (Q1, Q3) SD BB: 0.040 (0.011, 0.083); RMSSD BB: 0.047 (0.015, 0.086); *p* < 0.001).

EEG data from 25 participants (12 with moonbird first) was considered appropriate for further analysis, having good signal quality for all recording phases. Significant differences in global EEG power were observed only when comparing the intervention phase to baseline for the moonbird device, with higher relative delta power (median difference (Q1, Q3): 0.044 (0.020, 0.112); *p* < 0.01) and lower relative alpha power (median difference (Q1, Q3): −0.047 (−0.089, −0.020); *p* < 0.01) (Fig. 7a; Table A.3). Global EEG connectivity was significantly higher in the theta frequency range for the post-intervention resting phase after moonbird compared to the baseline (median difference (Q1, Q3): 0.050 (0.010, 0.072); *p* < 0.01) (Fig. 7b; Table A.4). The difference between devices for this same comparison, as a result of the Core device instead inducing a decrease in global theta connectivity, failed to retain significance following correction for multiple testing. No significant differences were found in EEG complexity as depicted by LZC (Table A.5).

**Fig. 7a:**
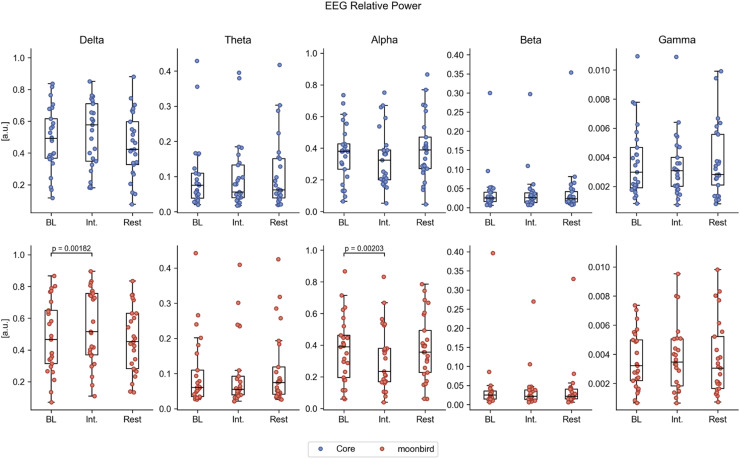
EEG relative power in the different frequency bands for the different phases and devices. BL = Baseline, int. = Intervention.

**Fig. 7b:**
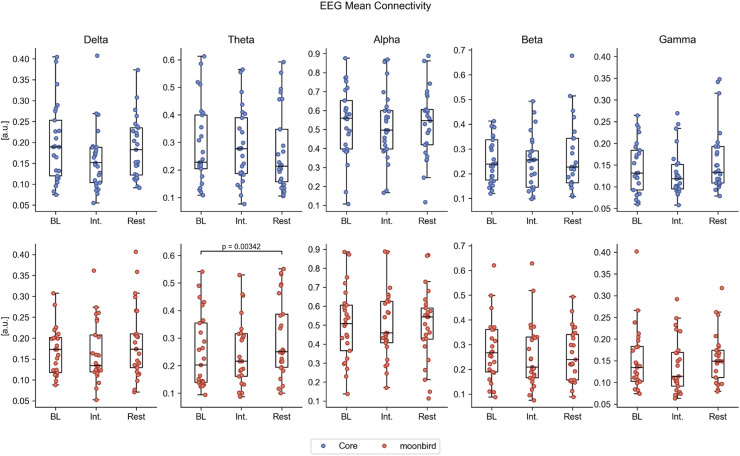
EEG mean connectivity in the different frequency bands for the different phases and devices. BL = Baseline, int. = Intervention.

### User preference

**Fig. 8:**
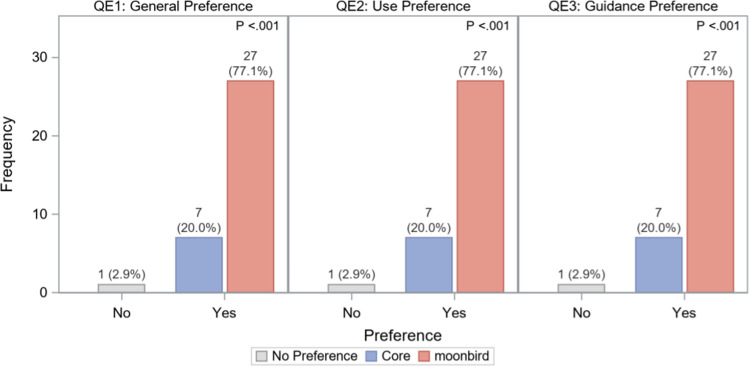
Questionnaire E: General device preference (QE1), Device holding preference (QE2), and Device guidance preference (QE3). *N* = 35.

There was a significant preference for the moonbird device compared to Core. Overall, 79 % of participants preferred moonbird (*p* < 0.001), and this preference increased to 94 % when moonbird was used second (Fig. 8). In terms of how the device should be held, moonbird was preferred by 79 % of participants (*p* < 0.001), rising to 100 % when it was used as the second device. Regarding breathing guidance (vibrations vs. expansion/contraction), moonbird (expansion/contraction) was favored in 79 % of instances (*p* < 0.001), with the preference going up to 89 % when it was the second device used.

### Psychological stress reduction

**Fig. 9:**
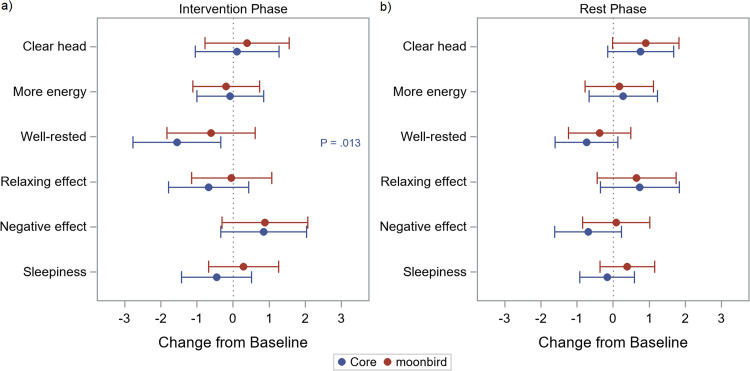
Questionnaire A: general experience – Change from baseline: (a) intervention phase minus baseline, and (b) post-intervention resting phase minus baseline.

The analysis of the effects of device-guided slow breathing on psychological stress (based on Questionnaire A) showed participants felt less rested during the use of the Core device (−1.56, CI: [−2.78; −0.34], Fig. 9a, Table A.6). However, after correcting for multiplicity this was not significant. Interestingly, participants seemed to have an improvement in mental clarity (*clear head*) in the post intervention rest phase compared to baseline for both devices, although this effect was not statistically significant. The other parameters (energy levels, relaxation effects, negative outcomes, sleepiness) did not vary (Fig. 9b; Table A.7).

**Fig. 10:**
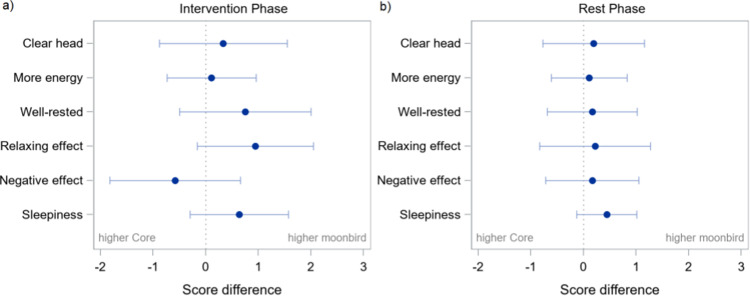
Questionnaire A: general experience – Estimated differences between devices (moonbird minus Core) and 95 % confidence intervals during intervention phase (a) and post-intervention phase (b).

When comparing the two devices regarding their impact on mental clarity, energy levels, feelings of restfulness, relaxation effects, negative outcomes, and sleepiness, no notable differences were observed for the intervention phase, or the post-intervention resting phase (Fig. 10; Table A.8, A.9), also not after adjusting for baseline measures.

**Fig. 11:**
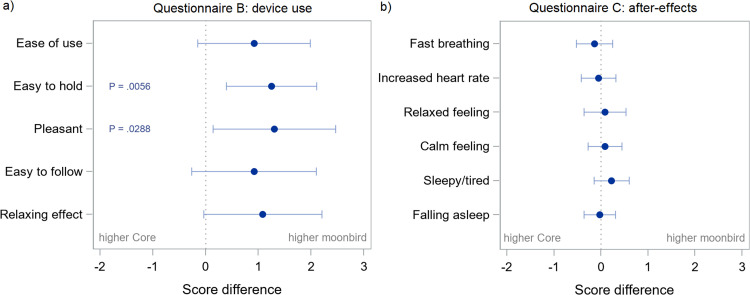
(a) Questionnaire B: device use and (b) Questionnaire C: after-effects – Estimated differences between devices (moonbird minus Core) and 95 % confidence intervals.

Ease of holding and the pleasantness of guidance were rated significantly higher for moonbird than for Core, with estimated differences and 95 % CI of 1.25 (0.39, 2.11) and 1.31 (0.14, 2.47), respectively (Fig. 11a). After correcting for multiplicity, only ease of holding remains statistically significant. In the sensitivity analysis, these differences were, however, not statistically significant (Table A.10). No significant differences between devices were found in terms of reported after-effects (rapid breathing, increased heartbeat, relaxed feeling, calm feeling, feeling sleepy and tired, falling asleep) (Fig. 11b; Table A.11).

### Associations between stress reduction effects and user preference

The analysis revealed possible associations between physiological stress reduction effects and self-reported device preference (Table 2; Fig. A.2, A.3). However, after correcting for multiplicity, none of these results remained significant and the results should be interpreted with care. RR was lower after using the preferred device compared to the non-preferred device (median difference (Q1, Q3): −0.79 (−2.00, −0.14); *p* < 0.01), also when adjusted for baseline (median difference (Q1, Q3): −1.22 (−2.15, 0.27); *p* < 0.01). Similarly, HR was lower during the use of the preferred device (median difference (Q1, Q3): −1.0 (−4.1, 0.1); *p* < 0.05). Beta connectivity was lower (median difference (Q1, Q3): −0.03 (−0.08, 0.00); *p* < 0.05), and theta connectivity, when corrected for baseline, was higher (median difference (Q1, Q3): 0.018 (−0.021, 0.093); *p* < 0.05), during the use of the preferred device. Additionally, beta power was lower during (median difference (Q1, Q3): −0.002 (−0.006, 0.001); *p* < 0.05) and after preferred device use (median difference (Q1, Q3): −0.002 (−0.007, 0.000); *p* < 0.05), while gamma power, when corrected for baseline, was higher during the use of the preferred device (median difference (Q1, Q3): 0.0008 (−0.0001, 0.0015); *p* < 0.05).

**Table 2:** Summary statistics (n, median and 1st and 3rd quartiles) and unadjusted p-values of the parameters with an unadjusted p-value <0.05 for the difference between the preferred and non-preferred device. The difference is the within-subject difference of the parameter for the preferred device minus the parameter of the non-preferred device. The p-values of these results come from Wilcoxon signed-rank test for paired samples. In case the data was not symmetrical enough (evaluated on histograms and by the Wilcoxon signed-rank test) the non-parametric Sign test for paired samples was used, indicated with an*.

Type of Preference	Parameter	Phase	Unadjusted p-value	Statistic	Non-Preferred	Preferred	Difference (Preferred minus Non-Preferred)
General (QE1)	θ connectivity	Device (adjusted for baseline)	0.0433*	n median (Q1, Q3)	25 0.002 (−0.038, 0.038)	25 0.028 (−0.053, 0.062)	25 0.018 (−0.021, 0.093)
	β Power	Rest	0.0488	n median (Q1, Q3)	25 0.022 (0.016, 0.034)	25 0.022 (0.014, 0.042)	25 −0.002 (−0.007, 0.000)
Use (QE2)	Respiratory rate	Rest	0.0015	n median (Q1, Q3)	24 14.8 (12.8, 17.1)	24 13.5 (10.6, 16.5)	24 −0.79 (−2.00, −0.14)
		Rest (adjusted for baseline)	0.0025	n median (Q1, Q3)	24 −0.48 (−1.95,0.83)	24 −2.23 (−4.26, −0.47)	24 −1.22 (−2.15, 0.27)
	β connectivity	Device	0.0271	n median (Q1, Q3)	25 0.256 (0.166, 0.328)	25 0.210 (0.162, 0.328)	25 −0.0314 (−0.0802, 0.0038)
	γ power	Device (adjusted for baseline)	0.0394	n median (Q1, Q3)	25 −0.0001 (−0.0012, 0.0004)	25 0.0000 (−0.0001, 0.0003)	25 0.0008 (−0.0001, 0.0015)
Guidance (QE3)	Heart Rate	Device	0.0433*	n median (Q1, Q3)	25 69.3 (62.0, 75.7)	25 66.8 (59.6, 71.2)	25 −1.0 (−4.1, 0.1)
	β Power	Device	0.0223	n median (Q1, Q3)	24 0.026 (0.013, 0.046)	24 0.022 (0.014, 0.039)	24 −0.002 (−0.006, 0.001)

## Discussion

This study aimed to evaluate the effectiveness of and user preferences for two breathing devices, moonbird and Core, designed to mitigate stress in individuals experiencing high stress levels. The investigation focused on physiological measures, user preferences, and psychological stress reduction to provide a comprehensive assessment of these devices.

No significant differences in mean heart rate were observed between devices. However, during device usage, HRV showed a significant increase in SD NN while ApEn significantly decreased. Most participants (80 %) managed to reduce their breathing rate close to the target set by the devices. However, only moonbird showed persistent effects on RRV post-intervention. The ratio of inspiratory to expiratory time remained unchanged, indicating that the devices primarily affected the breathing rate rather than the breathing rhythm. Moonbird usage was associated with EEG patterns indicative of modifications in relaxation and cognitive processing states (increased delta power, decreased alpha power) and enhanced connectivity in brain regions involved in stress regulation (increased theta connectivity post-intervention. There was a significant preference for the moonbird device over Core, particularly regarding handling and breathing guidance. Despite the strong preference, the sensitivity analysis did not confirm significant differences in ease of use and pleasantness. The preference for moonbird increased when it was used as the second device, suggesting possible order effects. Improvements in mental clarity were reported post-intervention for both devices. No notable differences were found between devices concerning other psychological outcomes (energy levels, relaxation effects, negative outcomes, sleepiness, absorption, dissociation, time perception). Possible associations were found between physiological stress reduction and self-reported device preference. Lower respiratory rate and heart rate were observed with the preferred device. Additionally, beta connectivity and beta power were reduced, while theta connectivity and gamma power increased during and after the use of the preferred device.

The lack of significant differences in heart rate between devices contrasts with the observed HRV changes. The increase in SD NN during device use suggests that both devices effectively influenced autonomic regulation, as SD NN reflects overall HRV and is indicative of parasympathetic activity. This finding aligns with similar studies where HRV increases during interventions aimed at stress reduction (Shao et al., 2024). The changes in frequency domain measures—higher LF and lower HF power—suggest a shift towards increased sympathetic dominance during device use, although this shift was temporary and did not persist post-intervention. The decrease in ApEn might indicate a more regular breathing pattern, consistent with the devices’ purpose to regulate breathing. This regularity might reflect the devices' effectiveness in guiding users towards a slower, more controlled breathing rate, which is known to have stress-reducing effects (Zaccaro et al., 2018).

The ability of most participants to reduce their breathing rate close to the target set by the devices supports the effectiveness of both moonbird and Core in guiding slow breathing. However, the fact that RRV effects persisted only for moonbird suggests that its feedback mechanism might be more effective in sustaining changes in breathing patterns. The unchanged inspiratory to expiratory time ratio indicates that while the devices succeeded in altering the breathing rate, they did not significantly modify the breathing rhythm. This is in line with the goal, within this study, to reduce the overall breathing rate rather than to alter breathing patterns in a more complex manner.

The increase in delta power and decrease in alpha power during moonbird use suggests a shift towards deeper relaxation or altered cognitive states. Delta power is often associated with deep sleep and restorative states, while alpha power is linked to relaxed wakefulness. The significant increase in theta connectivity post-intervention with moonbird reflects enhanced communication between brain regions involved in emotional and cognitive processing, which may contribute to stress reduction. The less pronounced changes observed with Core could be due to its different feedback mechanism or less pronounced effect on relaxation. The specific role of theta connectivity in stress reduction is supported by literature indicating its involvement in emotional regulation and cognitive processes (Knyazev, 2007).

The lack of significant differences in self-reported effects between the devices could be due to their similar mechanisms, as both use tactile guidance to promote slow breathing. Additionally, both devices may have been effective enough to reach a ceiling effect, making it difficult to discern differences. A placebo effect could also be at play, where participants’ belief in the devices’ effectiveness influences their reported outcomes. Lastly, individual differences in perception of relaxation and stress reduction may have contributed to the lack of significant differences, as personal biases and preconceptions can vary widely.

The significant preference for moonbird could be attributed to its ergonomic design and the nature of its breathing guidance. Its tactile feedback (expansion/contraction) may provide a more intuitive and satisfying user experience compared to Core’s vibration-based feedback. A device requiring two hands might be seen as less convenient for prolonged use. In the realm of haptic feedback technology (like in virtual reality controllers or mobile phones), vibrations are commonly used to provide feedback because they can be quickly and easily perceived. Devices that expand and contract might be used in situations where more pronounced feedback is needed or where the feedback represents specific information (like a breathing rhythm). The order effect observed—where the preference for moonbird increased when used second—suggests that participants’ experiences with each device may have influenced their preferences. Participants may rate the second device more favorably if it offers a perceived improvement compared to the first. This finding highlights the importance of considering order effects in device evaluations.

Exploratory analyses suggested a possible association between device preference and physiological stress markers, including lower respiratory rate, lower heart rate, and EEG changes such as increased theta connectivity and gamma power during use of the preferred device. This may indicate that user preference could enhance relaxation and stress reduction, possibly through increased comfort or perceived effectiveness. However, alternative explanations must be considered. The observed differences may partly reflect order effects or familiarization, as participants showed an increased preference for the moonbird device when it was used second. Placebo effects could also play a role, with participants' beliefs about a device's efficacy influencing both subjective experiences and physiological outcomes. It is important not to over-interpret these results due to several considerations. First, the extensive testing involved—14 parameters across four phases and three preference questions—results in a large total of tests. This raises the likelihood of obtaining significant p-values by chance. Second, there is a lack of consistency in the results when varying the preference question. For example, while significant differences in respiratory rate are observed when comparing preferred devices based on questionnaire E2 (use), such differences are not significant for E1 or E3. These factors necessitate cautious interpretation of the findings.

While our results show some clear physiological improvements (e.g., increased HRV, EEG changes in delta and theta activity), subjective reports of relaxation and stress reduction were less consistently significant. Several factors may explain this. First of all, low statistical power due to the relatively small sample size could be a contributor. Second, ceiling effects could have limited the sensitivity of self-report measures. Third, the short duration of the interventions may not have allowed enough time for conscious shifts to occur or be reported. Additionally, expectancy effects and conditioning could account for the physiological changes. Some participants may also have reported their experience retrospectively in ways that don’t align precisely with in-the-moment data ("delayed-recency" effect). Despite limited statistical significance in subjective outcomes, consistent trends — such as increased delta power, decreased alpha power, and higher relaxation scores post-intervention (e.g., Questionnaire C) — suggest an underlying alignment between physiological and psychological responses.

### Implications

For developers, this study underscores the importance of user-centric design in health-related devices. Devices should not only be effective but also comfortable and engaging. An exploratory hypothesis arising from this study, which warrants further investigation, is that positive psychological effects induced by user preferences could potentially influence and enhance physiological stress responses. Comparative testing can help refine designs based on user feedback and ensure that devices meet both functional and ergonomic needs. Clinicians should consider both the effectiveness and user experience when recommending breathing devices, while recognizing that perceived usability and comfort may influence outcomes. A device that aligns well with user preferences may enhance adherence and overall satisfaction, potentially leading to better stress management outcomes. End-users are encouraged to assess devices based on both immediate and long-term experiences, as personal fit can be a critical factor in sustained use and benefit.

### Limitations

This study faced several limitations. The lack of participant or assessor blinding may have introduced bias, particularly regarding user preferences and subjective outcomes. The predominant student sample—mostly young adults with similar socio-demographic backgrounds—limits the generalizability of the findings to broader, more diverse populations. Technical and material issues affected data quality and reduced the amount of analyzable data, and non-adherence of 20 % of participants to the breathing rhythms may have impacted the results. Additionally, the study lacked a control or placebo condition, such as a sham device or a breathing task without tactile feedback. Consequently, we cannot conclusively attribute the observed physiological changes solely to device-specific features, as placebo effects or general relaxation responses to guided breathing and music may have contributed. Although no non-intervention control group was included, the within-subject cross-over design allowed each participant to serve as their own control, helping to reduce inter-individual variability. Another limitation was the relatively small sample size that may have limited statistical power, particularly for detecting smaller effects. Finally, the large number of hypotheses tested increases the risk of Type I errors, although we applied Bonferroni corrections to mitigate this.

### Future directions

Future research should explore longitudinal effects of breathing devices and the evolution of user preferences over time. This could involve tracking changes in user satisfaction and efficacy over extended periods to provide a more comprehensive understanding of long-term effectiveness. Additionally, gathering real-time feedback from users in naturalistic settings can offer more relevant insights into actual usage patterns and preferences. Further investigations should also delve into the specific mechanisms behind EEG changes and their implications for stress reduction. Understanding these mechanisms could help refine device designs and enhance their effectiveness. Given the relatively small sample size in the present study, future research should also aim to replicate findings with larger and more heterogeneous samples and incorporate appropriate control conditions to better isolate device-specific effects. This would strengthen the robustness of conclusions. In addition, future studies could apply graph-theoretical measures, such as network integration and segregation, to uncover more subtle changes in functional connectivity beyond those captured by our current analyses.

The findings also imply the necessity for further research to comprehensively explore how user preference could impact physiological and psychological outcomes. Future studies should incorporate both subjective and objective measures to fully understand a potential interaction between device preference and stress reduction. Additionally, the results suggest that user experience factors, such as comfort and perceived effectiveness, may play an important role in how users engage with stress management devices. Designing devices that align with user preferences and expectations could potentially enhance their usability and perceived effectiveness, although further research is needed to determine their impact on physiological outcomes.

## Conclusion

This study compared two breathing devices, moonbird and Core, in their ability to reduce stress and for user preference. Both devices demonstrated physiological effects associated with stress reduction, with improvements in HRV and respiratory rate during use. Moonbird additionally induced more pronounced EEG changes linked to enhanced relaxation and cognitive regulation compared to Core. Participants favored moonbird for its ergonomic design and tactile feedback, which influenced their preference. This preference highlights the importance of device comfort and usability in stress management interventions. Future research should focus on the long-term effectiveness of these devices, incorporating real-world user feedback and examining the physiological and psychological mechanisms driving stress reduction. These insights will be critical for refining device design and improving their application in stress management.

## Author's contribution

EH, SB, CM and OG conceptualized the study. Survey selection was done by EH, CM and OG. Data collection and extraction were performed by EH. Data analyses were conducted by MM and LV. VO and JA provided support through expertise for analyses involving HRV/RRV and EEG, respectively. All authors contributed to the article, had full access to all the data in the study and had final responsibility for the decision to submit for publication.

## Conflict of interest

SB is co-founder of, and EH was, during the conduct of the study, researcher at Moonbird© BV. The remaining authors declare that the research was conducted in the absence of any commercial or financial relationships that could be construed as a potential conflict of interest. We declare no competing interests.

During the preparation of this work the author(s) used ChatGPT in order to improve fluency and readability. After using this tool/service, the author(s) reviewed and edited the content as needed and take(s) full responsibility for the content of the publication.

## Declaration of competing interest

The authors declare the following financial interests/personal relationships which may be considered as potential competing interests: Elisabeth Honinx reports financial support was provided by Flanders Innovation & Entrepreneurship. Elisabeth Honinx, Stefanie Broes reports a relationship with moonbird BV that includes: employment. If there are other authors, they declare that they have no known competing financial interests or personal relationships that could have appeared to influence the work reported in this paper.

## Appendix Supplementary materials

Image, application 1
