# Supplementary material for: The effectiveness and user preferences of two tactile breathing devices in reducing stress in stressed individuals: A mixed methods study
Source: Int J Clin Health Psychol. 2025 Jul 9;25(3):100603. doi: 10.1016/j.ijchp.2025.100603 (PMC12343360; doi:10.1016/j.ijchp.2025.100603)
Supplement: Supplementary file 1 [file mmc1.docx]

Appendix

|  | baseline | | intervention | | rest | |
| --- | --- | --- | --- | --- | --- | --- |
|  | Core | moonbird | Core | moonbird | Core | moonbird |
| Heart Rate  [beats/min] | 65.11  [60.53-72.78] | 67.82  [60.61-72.94] | 67.97  [60.79-74.33] | 65.30  [59.64-72.06] | 66.29  [58.46-70.21] | 64.34  [60.15-69.80] |
| HRV SD NN  [ms] | 68.03  [55.87-89.65] | 64.18  [56.77-85.76] | 103.70  [77.73-136.64] | 113.90  [80.86-130.53] | 85.51  [59.26-102.64] | 81.22  [60.95-105.44] |
| HRV RMSSD NN [ms] | 64.71  [44.94-77.79] | 59.62  [39.39-73.51] | 68.77  [47.86-90.51] | 71.09  [48.16-93.49] | 65.95  [39.49-74.11] | 69.63  [48.13-80.04] |
| HRV pNN50  [%] | 45.09  [23.80-51.46] | 35.97  [17.00-50.69] | 32.16  [20.47-42.09] | 35.83  [25.81-40.65] | 41.07  [18.30-46.75] | 41.41  [26.42-52.38] |
| HRV LFn  [a.u.] | 0.31  [0.23-0.36] | 0.30  [0.22-0.42] | 0.67  [0.56-0.74] | 0.69  [0.52-0.80] | 0.38  [0.25-0.51] | 0.31  [0.25-0.45] |
| HRV HFn  [a.u.] | 0.43  [0.33-0.50] | 0.35  [0.29-0.56] | 0.20  [0.16-0.25] | 0.19  [0.12-0.26] | 0.32  [0.20-0.44] | 0.33  [0.24-0.50] |
| HRV LF/HF  [a.u.] | 0.68  [0.47-1.19] | 0.79  [0.42-1.31] | 3.57  [2.28-4.16] | 3.69  [2.63-6.57] | 1.32  [0.59-2.61] | 0.90  [0.55-2.21] |
| HRV ApEn  [a.u.] | 1.33  [1.23-1.40] | 1.30  [1.22-1.40] | 1.04  [0.94-1.19] | 1.05  [0.94-1.14] | 1.25  [1.21-1.35] | 1.25  [1.18-1.33] |

**Table A.1: Summary of ECG-derived measures. (median [Q1-Q3])**

|  | baseline | | intervention | | rest | |
| --- | --- | --- | --- | --- | --- | --- |
|  | Core | moonbird | Core | moonbird | Core | moonbird |
| Respiratory Rate  [breaths/min] | 15.11  [12.98-17.72] | 16.96  [12.43-17.91] | 5.20  [5.08-6.15] | 5.19  [5.09-6.19] | 14.06  [11.42-16.71] | 13.45  [11.23-16.77] |
| PDR  [a.u.] | 0.80  [0.72-0.86] | 0.74  [0.71-0.83] | 1.05  [0.73-1.18] | 0.81  [0.64-1.01] | 0.81  [0.72-0.90] | 0.83  [0.72-0.92] |
| RRV SD BB  [ms] | 887.17  [599.52-1133.80] | 645.88  [455.90-841.00] | 2103.13  [1851.89-2913.58] | 1889.76  [1536.63-2560.12] | 1115.71  [661.34-1660.38] | 1000.52  [637.99-1292.22] |
| RRV RMSSD BB  [ms] | 871.00  [743.42-1293.69] | 704.94  [595.99-967.87] | 3028.95  [2689.06-3739.37] | 2930.60  [1995.15-3303.95] | 1082.44  [755.58-1691.98] | 1106.23  [814.78-1330.71] |

**Table A.2: Summary of respiration-derived measures. (median [Q1-Q3])**

|  | baseline | | intervention | | rest | |
| --- | --- | --- | --- | --- | --- | --- |
|  | Core | moonbird | Core | moonbird | Core | moonbird |
| delta | 0.4924 [0.3668-0.6166] | 0.4662 [0.3149-0.6502] | 0.5778 [0.3480-0.7107] | 0.5153 [0.3691-0.7559] | 0.4230 [0.3270-0.5975] | 0.4533 [0.2830-0.6305] |
| theta | 0.0750 [0.0387-0.1094] | 0.0605 [0.0346-0.1096] | 0.0561 [0.0395-0.1331] | 0.0549 [0.0392-0.0920] | 0.0620 [0.0393-0.1510] | 0.0745 [0.0415-0.1187] |
| alpha | 0.3813 [0.2677-0.4273] | 0.3897 [0.1956-0.4625] | 0.3249 [0.2001-0.3882] | 0.2344 [0.1685-0.3803] | 0.3892 [0.2707-0.4700] | 0.3562 [0.2272-0.4939] |
| beta | 0.0256 [0.0162-0.0405] | 0.0253 [0.01440.0353] | 0.0258 [0.0133-0.0389] | 0.0216 [0.0132-0.0385] | 0.0233 [0.0148-0.0419] | 0.0215 [0.0152-0.0404] |
| gamma | 0.0030 [0.0019-0.0047] | 0.0032 [0.0022-0.0050] | 0.0031 [0.0020-0.0040] | 0.0035 [0.0018-0.0051] | 0.0028 [0.0021-0.0056] | 0.0030 [0.0016-0.0052] |

**Table A.3: Summary of EEG relative power. (median [Q1-Q3])**

|  | baseline | | intervention | | rest | |
| --- | --- | --- | --- | --- | --- | --- |
|  | Core | moonbird | Core | moonbird | Core | moonbird |
| delta | 0.1890 [0.1198-0.2532] | 0.1727 [0.1176-0.2013] | 0.1515 [0.1040-0.1883] | 0.1344 [0.1192-0.2074] | 0.1826 [0.1222-0.2350] | 0.1736 [0.1293-0.2102] |
| theta | 0.2280 [0.2041-0.3998] | 0.2021 [0.1390-0.3545] | 0.2771 [0.1866-0.3897] | 0.2159 [0.1615-0.3161] | 0.2134 [0.1572-0.3479] | 0.2502 [0.1944-0.3869] |
| alpha | 0.5591 [0.3969-0.6521] | 0.5086 [0.3657-0.6053] | 0.4963 [0.3965-0.5998] | 0.4594 [0.4074-0.6251] | 0.5466 [0.4191-0.6055] | 0.5431 [0.4260-0.5903] |
| beta | 0.2386 [0.1743-0.3375] | 0.2668 [0.1895-0.3611] | 0.2559 [0.1454-0.2924] | 0.2096 [0.1662-0.3310] | 0.2272 [0.1630-0.3433] | 0.2399 [0.1576-0.3405] |
| gamma | 0.1312 [0.0925-0.1841] | 0.1343 [0.1027-0.1829] | 0.1182 [0.0950-0.1512] | 0.1141 [0.0915-0.1691] | 0.1330 [0.1086-0.1925] | 0.1488 [0.1113-0.1739] |

**Table A.4: Summary of EEG mean connectivity. (median [Q1-Q3])**

|  | baseline | | intervention | | rest | |
| --- | --- | --- | --- | --- | --- | --- |
|  | Core | moonbird | Core | moonbird | Core | moonbird |
| broadband | 0.4011 [0.3743-0.4477] | 0.4290 [0.3755-0.4459] | 0.4068 [0.3698-0.4383] | 0.4286 [0.3766-0.4501] | 0.4102 [0.3721-0.4430] | 0.4031 [0.3647-0.4469] |

**Table A.5: Summary of EEG mean complexity. (median [Q1-Q3])**

|  | **Main Analysis** | | **Sensitivity Analysis on Period 1** | |
| --- | --- | --- | --- | --- |
|  | **Estimate of the mean change from baseline + 95%CI** | | **Median change from baseline (P-value)** | |
| **Question** | **Core** | **moonbird** | **Core** | **moonbird** |
| QA3 clearhead | 0.111 [-1.053; 1.275] | 0.389 [-0.775; 1.553] | 1.0 (0.2101) | 0.5 (1.0000) |
| QA4 energy | -0.083 [-1.009; 0.842] | -0.194 [-1.120; 0.731] | 0.5 (0.6072) | 0.0 (0.7744) |
| QA5 well rested | -1.556 [-2.774; -0.337] * | -0.611 [-1.829; 0.607] | 0.0 (1.0000) | 0.5 (1.0000) |
| QA6 relaxing | -0.681 [-1.788; 0.427] | -0.042 [-1.149; 1.066] | 0.5 (0.8036) | 0.0 (1.0000) |
| QA7 negative | 0.847 [-0.341; 2.035] | 0.875 [-0.313; 2.063] | 0.0 (1.0000) | 0.5 (0.1460) |
| QA9 sleepiness | -0.458 [-1.432; 0.515] | 0.292 [-0.682; 1.265] | 0.0 (1.0000) | 1.0 (0.3323) |
| **Table A.6. Questionnaire A - Change from baseline during intervention phase.** This table shows the estimated change from baseline during the intervention phase and 95% CI per device after correcting for (possible) period effect. These estimates come from a mixed effects model with change from baseline as a dependent variable and Period and Device as fixed effects, with random effect for subject. | | | | |
|  | | | | |

|  | **Main Analysis** | | **Sensitivity Analysis on Period 1** | |
| --- | --- | --- | --- | --- |
|  | **Estimate of the mean change from baseline + 95%CI** | | **Median change from baseline (P-value)** | |
| **Question** | **Core** | **moonbird** | **Core** | **moonbird** |
| QA3 clearhead | 0.764 [-0.153; 1.681] | 0.903 [-0.014; 1.819] | 1.0 (0.2379) | 0.5 (0.1460) |
| QA4 energy | 0.278 [-0.668; 1.223] | 0.167 [-0.779; 1.112] | 0.5 (0.8036) | 0.5 (0.8036) |
| QA5 well rested | -0.736 [-1.603; 0.131] | -0.375 [-1.242; 0.492] | -0.5 (0.2668) | 0.0 (1.0000) |
| QA6 relaxing | 0.736 [-0.355; 1.827] | 0.653 [-0.438; 1.744] | 0.0 (0.7905) | 1.0 (0.2101) |
| QA7 negative | -0.694 [-1.623; 0.234] | 0.083 [-0.845; 1.011] | 0.0 (0.1250) | 0.0 (1.0000) |
| QA9 sleepiness | -0.167 [-0.922; 0.588] | 0.389 [-0.366; 1.144] | 0.0 (0.5811) | 1.0 (0.1185) |
| **Table A.7. Questionnaire A - Change from baseline during post-intervention resting phase.** This table shows the estimated change from baselines during post-intervention rest phase and 95% CI per device after correcting for (possible) period effect. These estimates come from a mixed effects model with change from baseline as a dependent variable and Period and Device as fixed effects, with random effect for subject. | | | | |

|  | **Main Analysis** | | | | **Sensitivity Analysis on Period 1** | | |
| --- | --- | --- | --- | --- | --- | --- | --- |
| **Question** | **Estimate moonbird - Core** | **95%CI** | | **P-value** | **Median moonbird - Median Core** | **Mean moonbird - Mean Core** | **P-value** |
| QA3 clear head | 0.3333 | [-0.8808; 1.5475] | | 0.5805 | -0.5 | -0.9444 | 0.3042 |
| QA4 energy | 0.1111 | [-0.7375; 0.9598] | | 0.7918 | 1 | -0.5000 | 0.7860 |
| QA5 well rested | 0.7500 | [-0.4995; 1.9995] | | 0.2309 | 0 | -0.5556 | 0.5636 |
| QA6 relaxing | 0.9444 | [-0.1594; 2.0483] | | 0.0911 | 1 | 0.0000 | 0.9618 |
| QA7 negative | -0.5833 | [-1.8247; 0.6581] | | 0.3463 | 1.5 | 0.8889 | 0.3343 |
| QA9 sleepiness | 0.6389 | [-0.2958; 1.5736] | | 0.1739 | 1 | -0.2778 | 0.7852 |
| The main analysis estimates the treatment difference of moonbird - Core, using the Hill's Armitage approach for cross-over studies. The sensitivity analysis only looks at Period 1, in case there is a carry-over (or Period*Treatment) effect. For this a non-Parametric Mann-Whitney U test is performed. We also show the differences (moonbird - Core) of the means and medians. N = 36. | | | | | | | |

**Table A.8. Questionnaire A - Comparison of devices during intervention phase**

|  | **Main Analysis** | | | | **Sensitivity Analysis on Period 1** | | |
| --- | --- | --- | --- | --- | --- | --- | --- |
| **Question** | **Estimate moonbird - Core** | **95%CI** | | **P-value** | **Median moonbird - Median Core** | **Mean moonbird - Mean Core** | **P-value** |
| QA3 clear head | 0.1944 | [-0.7695; 1.1584] | | 0.6844 | 0 | -0.2222 | 0.9480 |
| QA4 energy | 0.1111 | [-0.6100; 0.8322] | | 0.7561 | -1 | -0.5556 | 0.4055 |
| QA5 well rested | 0.1667 | [-0.6901; 1.0234] | | 0.6951 | 0 | -0.6111 | 0.4780 |
| QA6 relaxing | 0.2222 | [-0.8321; 1.2766] | | 0.6711 | -1 | -0.4444 | 0.5537 |
| QA7 negative | 0.1667 | [-0.7166; 1.0499] | | 0.7038 | 0 | 1.1111 | 0.1624 |
| QA9 sleepiness | 0.4444 | [-0.1250; 1.0139] | | 0.1220 | 1 | 0.5556 | 0.2156 |
| The main analysis estimates the treatment difference of moonbird - Core, using the Hill's Armitage approach for cross-over studies. The sensitivity analysis only looks at Period 1, in case there is a carry-over (or Period*Treatment) effect. For this a non-Parametric Mann-Whitney U test is performed. We also show the differences (moonbird - Core) of the means and medians. N = 36 | | | | | | | |

**Table A.9. Questionnaire A - Comparison of devices during post-intervention resting phase**

|  | **Main Analysis** | | | | **Sensitivity Analysis on Period 1** | | |
| --- | --- | --- | --- | --- | --- | --- | --- |
| **Question** | **Estimate moonbird - Core** | **95%CI** | | **P-value** | **Median moonbird - Median Core** | **Mean moonbird - Mean Core** | **P-value** |
| QB1 ease of use | 0.9167 | [-0.1471; 1.9805] | | 0.0889 | 0 | -0.3333 | 0.7224 |
| QB2 ease to hold | 1.2500 | [0.3914; 2.1086] | | 0.0056 | 0 | 0.0556 | 0.9485 |
| QB3 guidance: pleasantness | 1.3056 | [0.1434; 2.4677] | | 0.0288 | 1 | 0.2778 | 0.7379 |
| QB4 guidance: ease to follow | 0.9167 | [-0.2625; 2.0958] | | 0.1234 | -1.5 | -0.7222 | 0.3268 |
| QB5 relaxing effect | 1.0833 | [-0.03811; 2.2048] | | 0.0578 | 0.5 | -0.1111 | 0.8224 |
| The main analysis estimates the treatment difference of moonbird - Core, using the Hill's Armitage approach for cross-over studies.The sensitivity analysis only looks at Period 1, in case there is a carry-over (or Period*Treatment) effect. For this a non-Parametric Mann-Whitney U test is performed. We also show the differences (moonbird - Core) of the means and medians. N = 36. Statistical significance is indicated with *. | | | | | | | |

**Table A.10. Questionnaire B - Comparison of devices during intervention**

|  | **Main Analysis** | | | | **Sensitivity Analysis on Period 1** | | |
| --- | --- | --- | --- | --- | --- | --- | --- |
| **Question** | **Estimate moonbird - Core** | **95%CI** | | **P-value** | **Median moonbird - Median Core** | **Mean moonbird - Mean Core** | **P-value** |
| QC1 fast breathing | -0.1389 | [-0.5252; 0.2474] | | 0.4697 | 0 | -0.1732 | 0.6248 |
| QC2 fast heartbeat | -0.05065 | [-0.4202; 0.3189] | | 0.7821 | 0 | -0.0523 | 0.9860 |
| QC6 relaxed feeling | 0.08007 | [-0.3656; 0.5257] | | 0.7171 | 0 | 0.2712 | 0.4158 |
| QC7 calm feeling | 0.08170 | [-0.2779; 0.4413] | | 0.6469 | 0 | 0.1209 | 0.8582 |
| QC8 sleepy tired | 0.2239 | [-0.1512; 0.5989] | | 0.2332 | 1 | 0.1209 | 0.7977 |
| QC9 falling asleep | -0.02614 | [-0.3581; 0.3058] | | 0.8737 | 0 | -0.1471 | 0.7718 |
| The main analysis estimates the treatment difference of moonbird - Core, using the Hill's Armitage approach for cross-over studies. The sensitivity analysis only looks at Period 1, in case there is a carry-over (or Period*Treatment) effect. For this a non-Parametric Mann-Whitney U test is performed. We also show the differences (moonbird - Core) of the means and medians. N = 36 | | | | | | | |

**Table A.11. Questionnaire C - Comparison of devices during post-intervention resting phase**

Figures


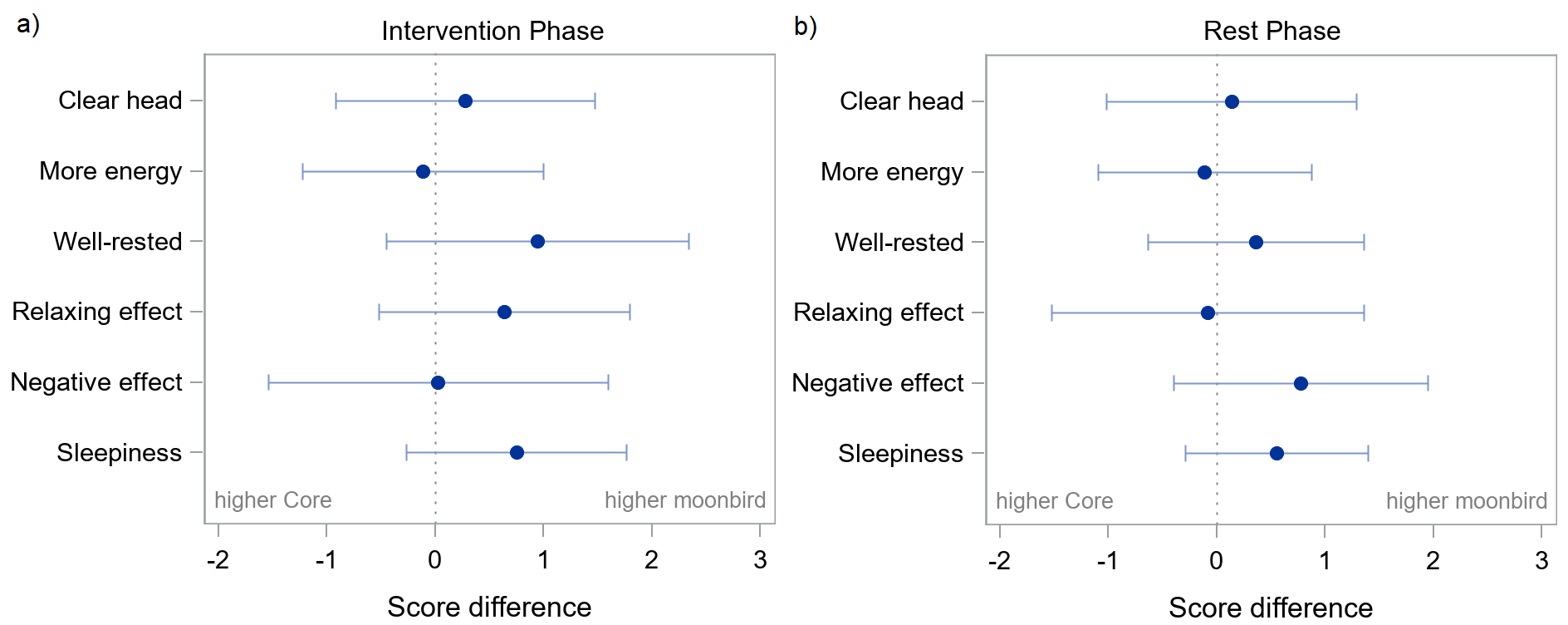


**Figure A.1. Questionnaire A: general experience -** Estimated differences between devices (moonbird minus Core) adjusted for baseline and 95% confidence intervals during intervention (a) and post-intervention phase (b).


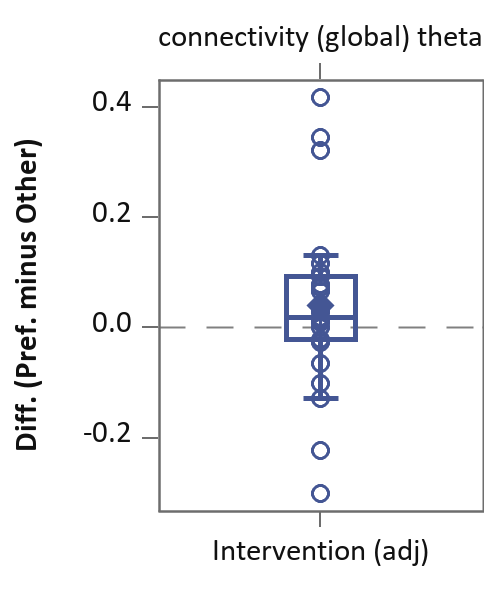

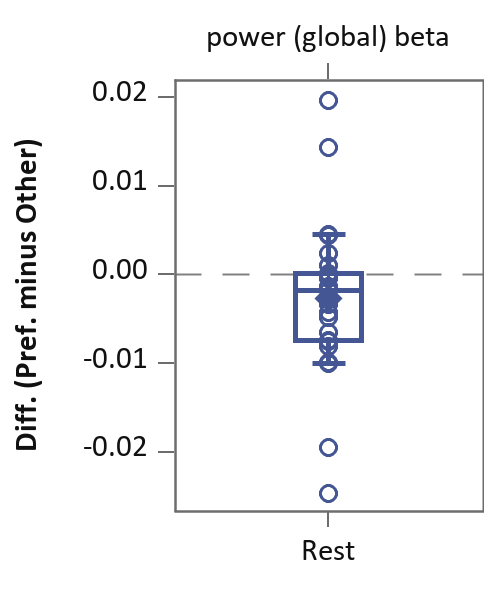


QE1 (General Preference)


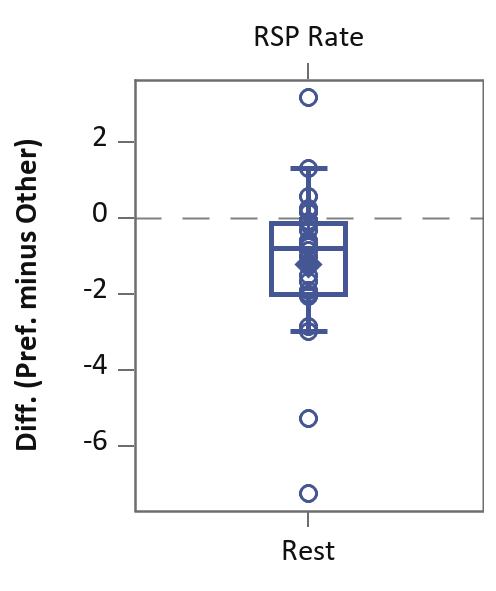

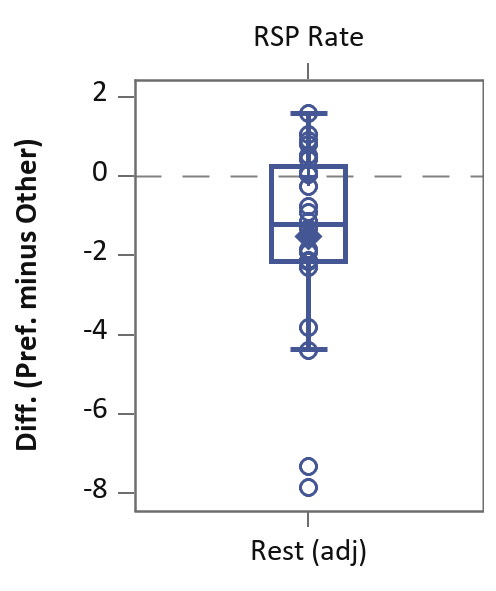

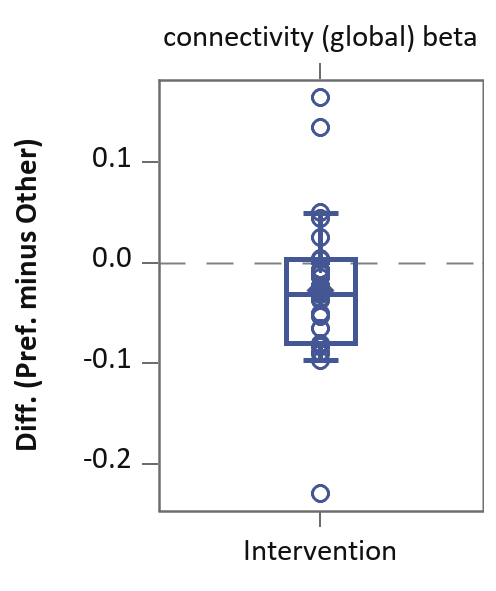

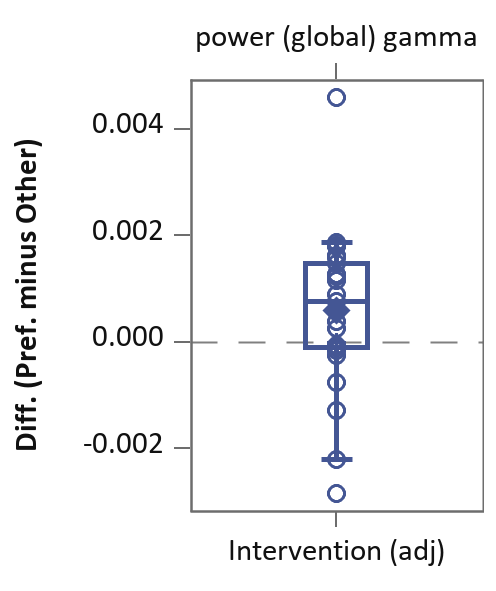


QE2 (Use Preference)


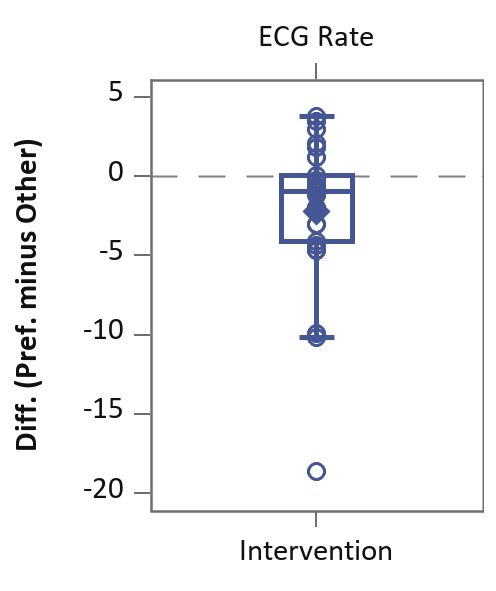

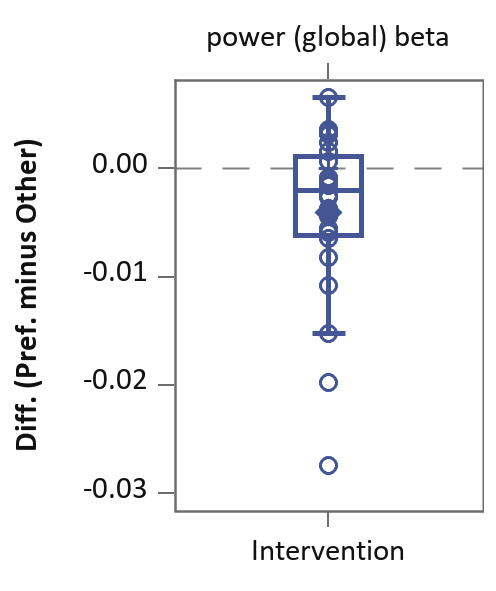


QE3 (Guidance Preference)

**Figure A.2: Boxplots of the within-subject differences (preferred device minus the other), for the significantly different results.** The results are presented by preference type, because some patients had a different preferred device depending on the question, e.g., there were patients that generally preferred moonbird, but preferred the guidance of the Core device. When values from the intervention phase, respectively rest phase, were adjusted for baseline before calculating the within-subject difference, this is presented in the plot as intervention (adj), respectively rest (adj).

QE1 (General preference)


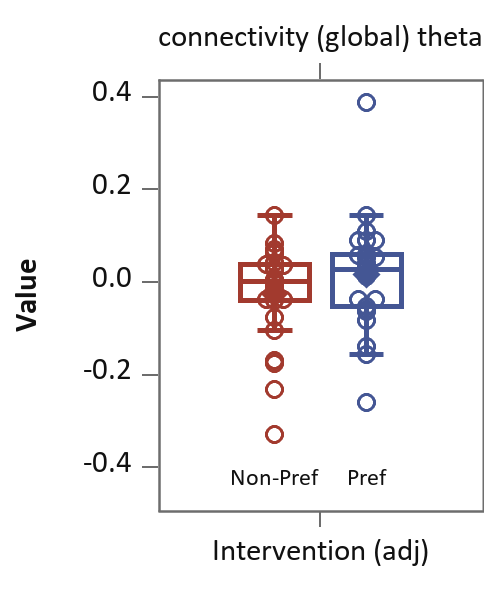

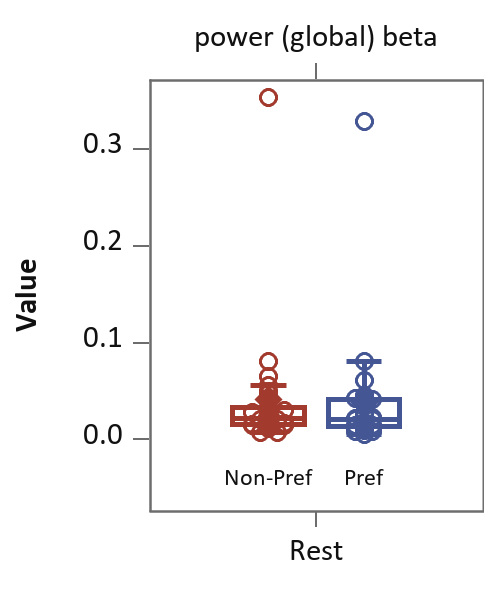


QE2 (Use Preference)


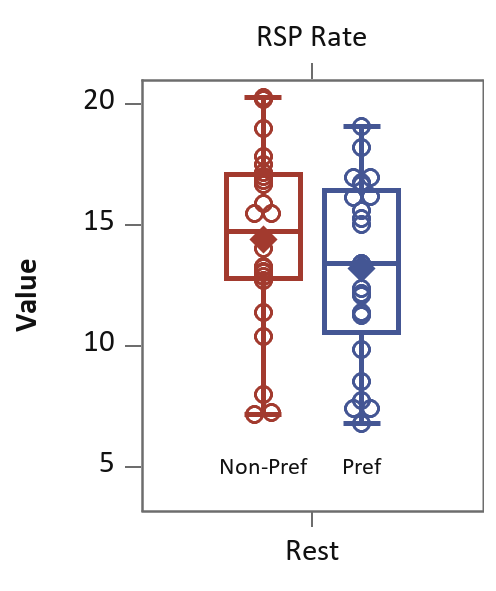

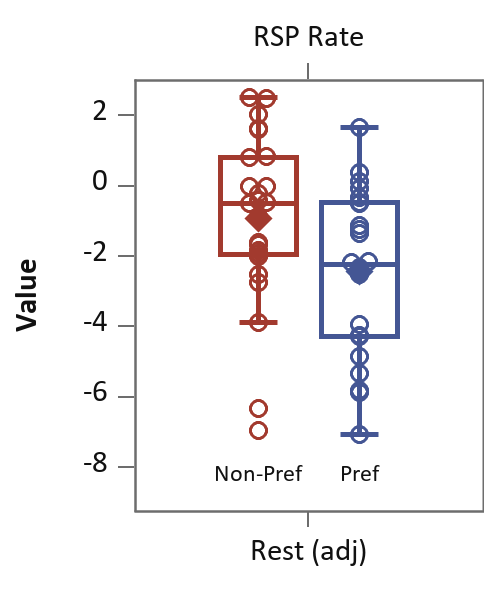

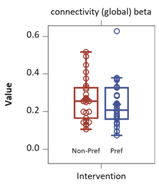

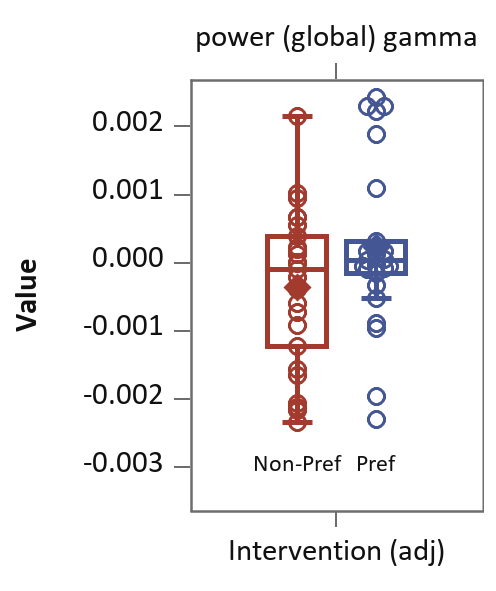


QE3 (Guidance preference)


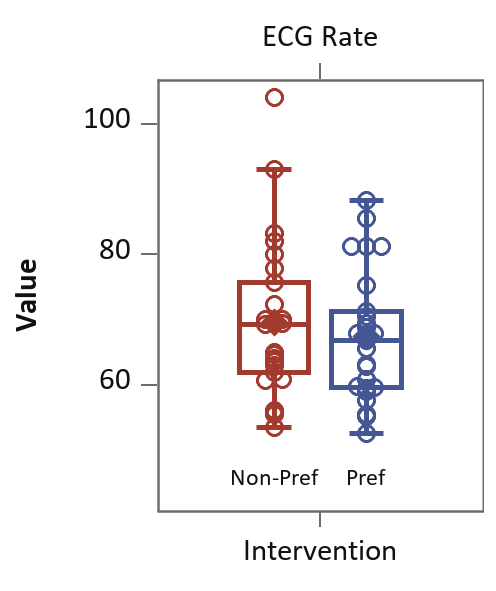

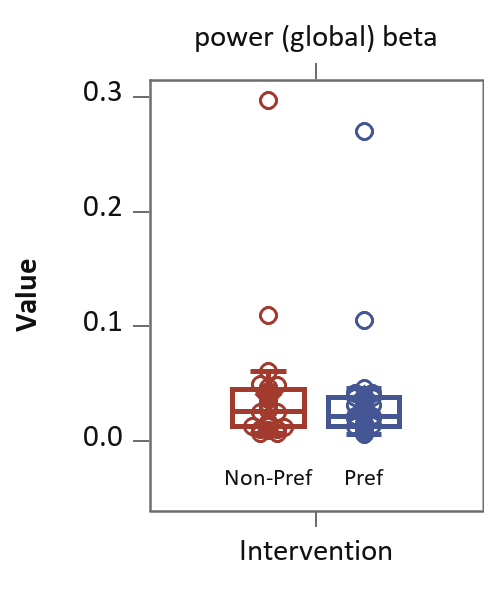


**Figure A.3: Boxplots of the parameter values by preferred/non-preferred device for the significantly different results.** The results are presented by preference type, because some patients had a different preferred device depending on the question, e.g., there were patients that generally preferred moonbird, but preferred the guidance of the Core device. When values from the intervention phase, respectively rest phase, were adjusted for baseline before calculating the within-subject difference, this is presented in the plot as intervention (adj), respectively rest (adj).
